# Differential expression of hyperpolarization-activated cyclic nucleotide-gated channel subunits during hippocampal development in the mouse

**DOI:** 10.1186/s13041-015-0103-4

**Published:** 2015-02-27

**Authors:** Hyunhyo Seo, Myoung-Jin Seol, Kyungmin Lee

**Affiliations:** Department of Anatomy, Brain Science & Engineering Institute, Kyungpook National University Graduate School of Medicine, 2-101, Dongin-dong, Jung-gu Daegu, 700-842 South Korea

**Keywords:** HCN channels, Hippocampus, Neurodevelopment, Dentate gyrus, Cornu Ammonis, Stratum lacunosum moleculare, Immunohistochemistry

## Abstract

**Background:**

Hyperpolarization-activated cyclic nucleotide-gated (HCN) channels help control the rhythmic activation of pacemaker neurons during brain development. However, little is known about the timing and cell type specificity of the expression of HCN isoforms during development of the hippocampus.

**Results:**

Here we examined the developmental expression of the brain-enriched HCN1, HCN2, and HCN4 isoforms of HCN channels in mouse hippocampus from embryonic to postnatal stages. All these isoforms were expressed abundantly in the hippocampus at embryonic day 14.5 and postnatal day 0. Each HCN channel isoform showed subfield-specific expression within the hippocampus from postnatal day 7, and only HCN4 was found in glial cells in the *stratum lacunosum moleculare* at this developmental stage. At postnatal days 21 and 56, all HCN isoforms were strongly expressed in the *stratum lacunosum moleculare* and the *stratum pyramidale* of the *Cornu Ammonis* (CA), as well as in the *hilus* of the *dentate gyrus*, but not in the subgranular zone. Furthermore, the immunolabeling for all these isoforms was colocalized with parvalbumin immunolabeling in interneurons of the CA field and in the *dentate gyrus*.

**Conclusions:**

Our mapping data showing the temporal and spatial changes in the expression of HCN channels suggest that HCN1, HCN2, and HCN4 subunits may have distinct physiological roles in the developing hippocampus.

## Background

Hyperpolarization-activated cyclic nucleotide-gated (HCN) channels are present in cardiac muscle cells [1] and in brain neurons [2]. In neurons, HCN channels are observed in dendrites [3,4], and in presynaptic axon terminals [5,6], where they have a role in regulating synaptic transmission [3,4]. These channels conduct a hyperpolarization-activated cation current (Ih), which plays a key role in the pacemaker depolarization that generates rhythmic activity, and mediates a variety of neural functions [7]. The properties of these currents passing through the HCN channels affect membrane excitability and the synchronized activity of neurons [8,9]. Four subunits (HCN 1–4) of HCN channels that conduct Ih currents have been identified [10-13]. HCN channels are assembled as homo- or heterotetramers of these subunits, which confer them specific biophysical characteristics. HCN1 channels are abundantly expressed in pyramidal neurons, where they are activated in a relatively rapid manner on hyperpolarization (tenths of milliseconds), showing minimal response to cAMP [14]. HCN2 channels are widely expressed throughout the brain, where they are activated more slowly (hundreds of milliseconds), and are strongly modulated by cAMP [11,15,16]. Finally, HCN4 channels are expressed in subcortical regions of the brain, where they are activated very slowly (seconds), and respond strongly to cAMP; while HCN3 channels show very low levels of expression in the brain [12,13,15]. The biophysical diversity of HCN channels suggests that differential gene expression and assemblage of the HCN subunits may produce the heterogeneity observed in Ih currents [17].

The dorsal hippocampus is considered an integral part of the brain circuitry involved in cognitive function. Anomalies in this brain region have been observed in several psychiatric disorders, including schizophrenia and anxiety, mood, and bipolar disorders [18-20], as well as in neurodevelopmental disorders such as epilepsy [21,22]. In addition, Ih currents participate in network activity in the developing hippocampus [23-25]. The distinct role of Ih currents, which exhibit age-specific biophysical properties in the developing hippocampus of rodents, has been attributed to the developmental molecular heterogeneity of HCN channels in the nervous system [9,26]. In fact, neonatal expression of HCN channels in the rodent brain [27-29] is quite different from that observed in adult hippocampus [17,30]. However, the identification of age-dependent patterns and specific cell type expression of different HCN channel subunits in the hippocampus has remained elusive.

In this study, we mapped the expression of the three HCN subunits that are strongly expressed in the brain (i.e., HCN1, HCN2, and HCN4), and assessed cell type and age-specific expression of these HCN channels in embryonic and postnatal hippocampus.

## Results

### Hippocampal expression of HCN channel subunits in the brain of E14.5 embryos

**Figure 1:**
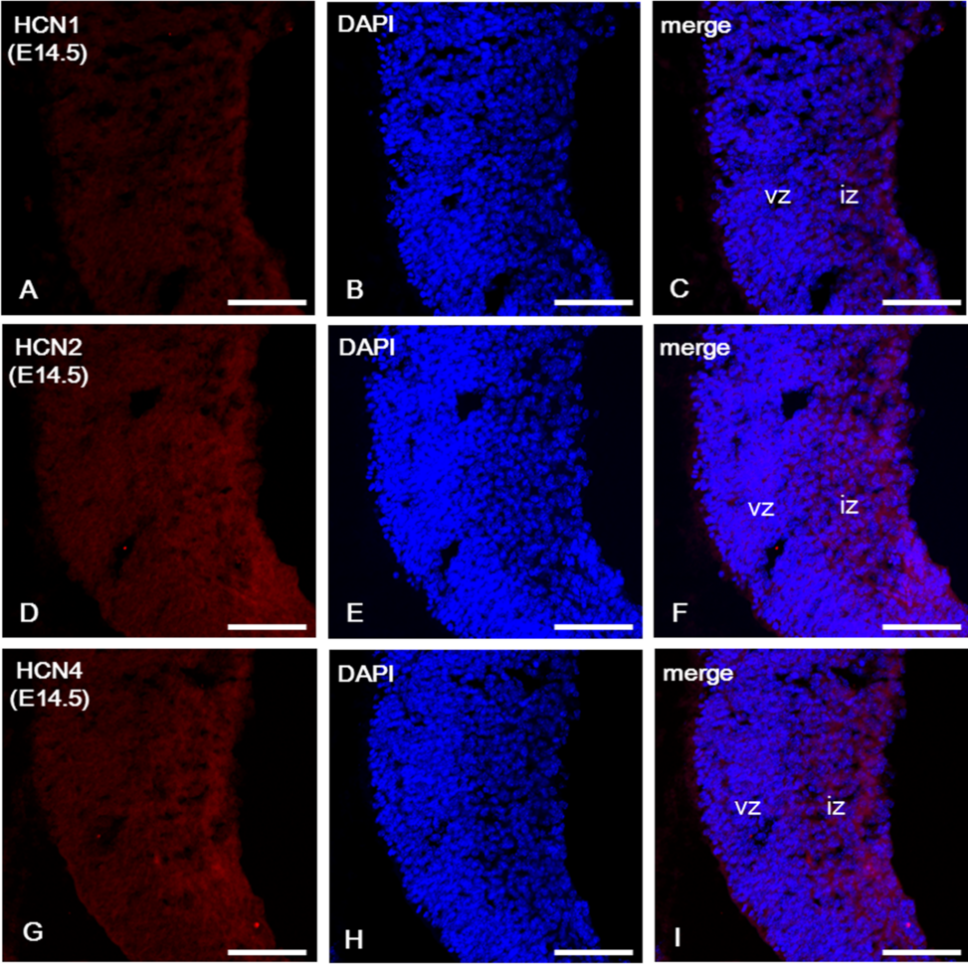
**Expression of HCN subunits in the hippocampal region at E14.5.** HCN1 **(A)**, HCN2 **(D)**, and HCN4 **(G)** immunolabeling was more evident in the intermediate zone (iz) than in the ventricular zone (vz). In the hippocampal iz, labeling for HCN2 and HCN4 subunits was higher than for HCN1. **B**, **E**, **H**: DAPI staining in the nucleus. **C**, **F**, **I**: merged images of HCN immunolabeling and DAPI stain. Scale bars = 20 μm.

The hippocampus originates from the dorsomedial telencephalon, which commences its invagination to build the medial walls of the telencephalic hemispheres at approximately E11 in the mouse. The differential gene patterning of the hippocampal region is apparent in the medial telencephalic wall at E14.5–E15.5, when the hippocampal fields begin to differentiate [31,32]. We investigated the expression of HCN channel subunits in the brain of E14.5 embryos. At E14.5, all HCN subunits were expressed in the hippocampal region, including the ventricular (VZ) and the intermediate (IZ) zones; their expression was more intense in the IZ than in the VZ (Figure 1). In addition, immunolabeling for HCN2 and HCN4 was generally stronger (Figure 1D-I) than for HCN1 (Figure 1A-C).

### Hippocampal expression of HCN channel subunits in the brain of P0 and P7 pups

**Figure 2:**
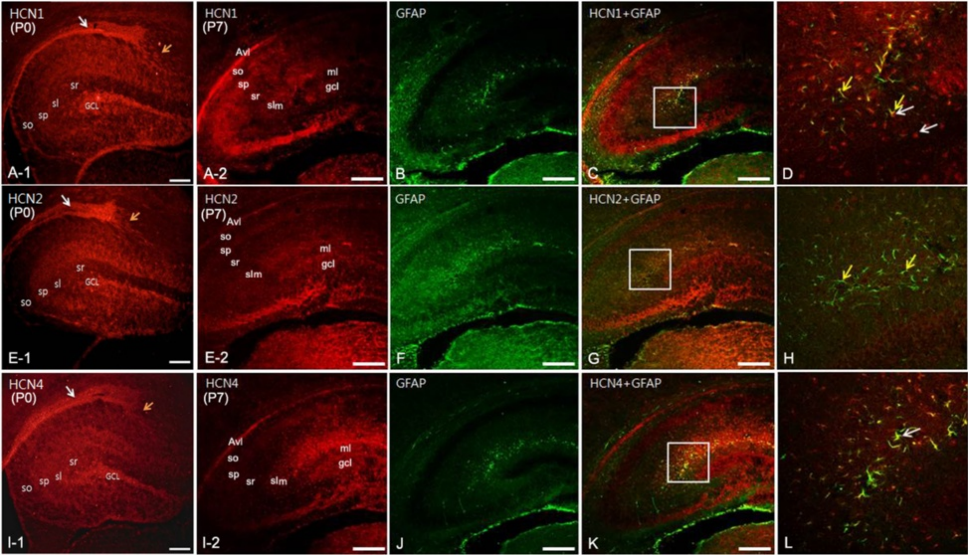
**Expression of HCN subunits in the hippocampus at P0 and P7. (A-1)**: At P0, the expression of HCN1 was strong in the *alveus* and *so*, *sp*, *sl*, and *sr* of the CA, and in the *GCL* of the *dentate gyrus* (DG). **(E-1)**: HCN2 expression at P0 was observed in the *alveus*, *sp*, *sl*, and *sr* of the CA, and in the *GCL* of the DG. **(I-1)**: HCN4 showed a similar pattern of expression to HCN1 at P0. Note that migrating cells (yellow arrows) from the ventricular zone (vz, white arrows) expressed all HCN isoforms, but the expression of HCN1 and HCN2 subunits was more prominent than that of HCN4. At P7, immunolabeling for HCN1 **(A-2)**, HCN2 **(E-2)**, and HCN4 **(I-2)** was observed in the *sp* and *slm* of the CA, and in the *gcl* of the DG. HCN1 **(B, C, D)** and HCN2 **(F, G, H)** subunits were expressed in neuronal somata, but not in astrocytes. Labeling for HCN4 was observed in the *slm*, as well as in the border of the *slm* with the molecular layer (*ml*) of the DG. **(B, F, J)**: Double immunofluorescence with GFAP showed that most GFAP-positive astrocytes were also immunolabeled with HCN4 in the *slm,* and in the border of the *ml* in the DG. **B**, **C**, **D**: yellow arrows indicate HCN1 labeling, white arrows indicate GFAP labeling. **F**, **G**, **H**: yellow arrows indicate HCN2 labeling. **J**, **K**, **L**: white arrow indicates an astrocyte double-labeled for HCN4 and GFAP. Abbreviations: GCL (or gcl), granule cell layer; sl, stratum lucidum; slm, stratum lacunosum moleculare; so, stratum oriens; sp, stratum pyramidale; sr, stratum radiatum. Scale bars = 20 μm.

At P0, the expression of HCN1 (Figure 2A-1) was observed in the *stratum oriens* (SO), the *stratum pyramidale* (SP), and the *stratum lucidum* (SL) of the *Cornu Ammonis* (CA). In contrast, immunoreactivity for HCN2 (Figure 2E-1) was strong in the SP and the SL, whereas HCN4 subunit (Figure 2I-1) showed a similar pattern of expression to HCN1. However, the *alveus* near the VZ (Figure 2A-1, E-1, C-1), and the *dentate gyrus,* exhibited the most intense expression for the three HCN subunits (Figure 2A-1, E-1, C-1). Hippocampal neurons originate from the ventricular neuroepithelium and the neuroepithelium adjacent to the fimbria. Here we found many cells migrating from the VZ to the hippocampus. Interestingly, these newly generated cells showed more intense labeling for HCN1 and HCN2 subunits than for HCN4 (Figures 2A-1, E-1, C-1).

At P7, the expression of all HCN subunits was remarkably decreased in the *alveus* compared to P0 (Figure 2A-2, E-2, I-2). Immunolabeling for HCN1 (Figure 2A-2), HCN2 (Figure 2E-2), and HCN4 (Figure 2I-2) subunits was observed in the SP and in the *stratum lacunosum-moleculare* (SL-M) of the CA, as well as in the granule cell layer (GCL), but not in the molecular layer (ML) of the *dentate gyrus*. Particularly, the expression of HCN4 was observed in the SL-M, and at the border of the SL-M with the ML of the *dentate gyrus* (Figure 2I-2, K). Since P7 is a period of astrocytogenesis, we performed double immunofluorescence with glial fibrillary acidic protein (GFAP) as a marker for astrocytes, to identify specific cell types that expressed each HCN subunit in the SL-M. We observed expression of HCN1 (Figure 2B-D) and HCN2 (Figure 2F-H) in neuronal somata (but not in astrocytes) of the SL-M, and in the border of the SL-M with the ML of the *dentate gyrus*. On the other hand, most GFAP-positive astrocytes were immunolabeled for HCN4 in the SL-M (Figure 2J-L), and in the border of the SL-M with the ML of the *dentate gyrus*.

### Hippocampal expression of HCN channel subunits in the juvenile brain at P21

**Figure 3:**
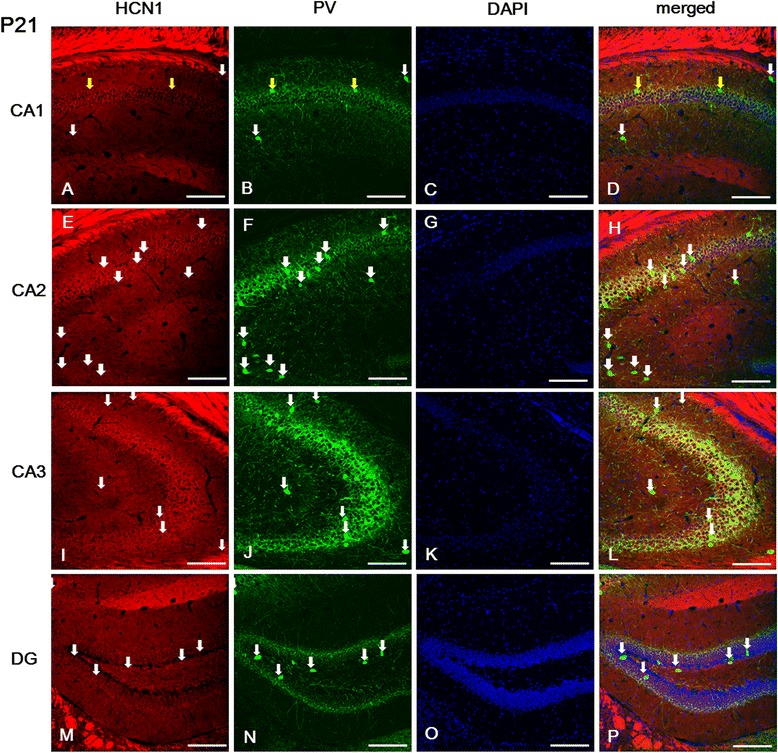
**Expression of HCN1 in parvalbumin (PV)-positive cells of the hippocampus at P21. (A, E, I)**: The strongest labeling for HCN1 was observed in the *sl-m* and *alveus* of the CA. Note also that its expression was gradually stronger in the *so, sp,* and *sr* of the CA2 **(E)** and CA3 **(I)** compared to CA1 **(A)**. **(A, B, D)**: PV-positive cells located in the *sp* of the CA1 did not show HCN1 immunolabeling, whereas PV-positive cells in the *so* or *sr* of the CA1 were labeled for HCN1. In the CA2, CA3, and *hilus* of the DG, most PV-immunopositive cells were labeled for HCN1. **A**-**B**, **D**: yellow arrows indicate PV-positive/HCN1-negative cells. **A**-**B**, **D**-**F**, **H**-**J**, **L**-**N**, **P**: white arrows indicate PV-positive/HCN1-positive cells. **C**, **G**, **K**, **O**: DAPI staining. Scale bars = 20 μm.

**Figure 4:**
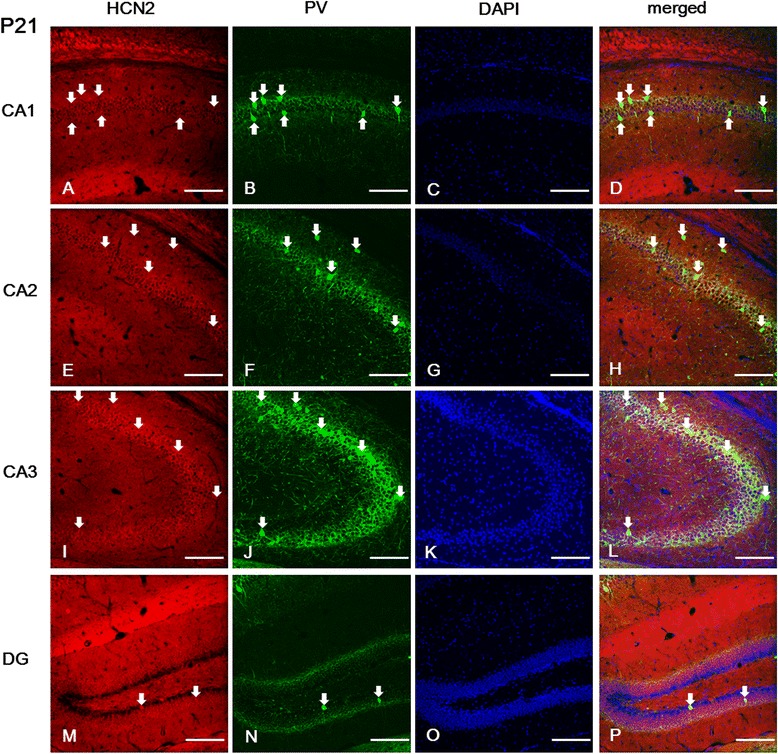
**Expression of HCN2 in PV-positive cells of the hippocampus at P21. (A-B, D-F, H-J, L-N, P)**: The images show HCN2 labeling in different fields of the hippocampus and the DG. **(A, E, I)**: Note that HCN2 expression was more prominent in the *sl-m* and *sp* of the CA, particularly in CA3. Interestingly, the *alveus* did not show HCN2 expression at this developmental stage. In addition, the *sl-m* and *sp* of the CA3 **(I)** presented stronger labeling than in the CA1 **(A)** and the CA2 **(E)**. Most PV-immunopositive cells located in the *sp* of the CA1 **(A, B, D)**, CA2 **(E, F, H)**, CA3 **(I, J, L)**, and *hilus* of the DG **(M, N, P)** co-expressed HCN2. **A**-**B**, **D**-**F**, **H**-**J**, **L**-**N**, **P**: white arrows indicate PV-positive/HCN2-positive cells. **C**, **G**, **K**, **O**: DAPI staining. Scale bars = 20 μm.

**Figure 5:**
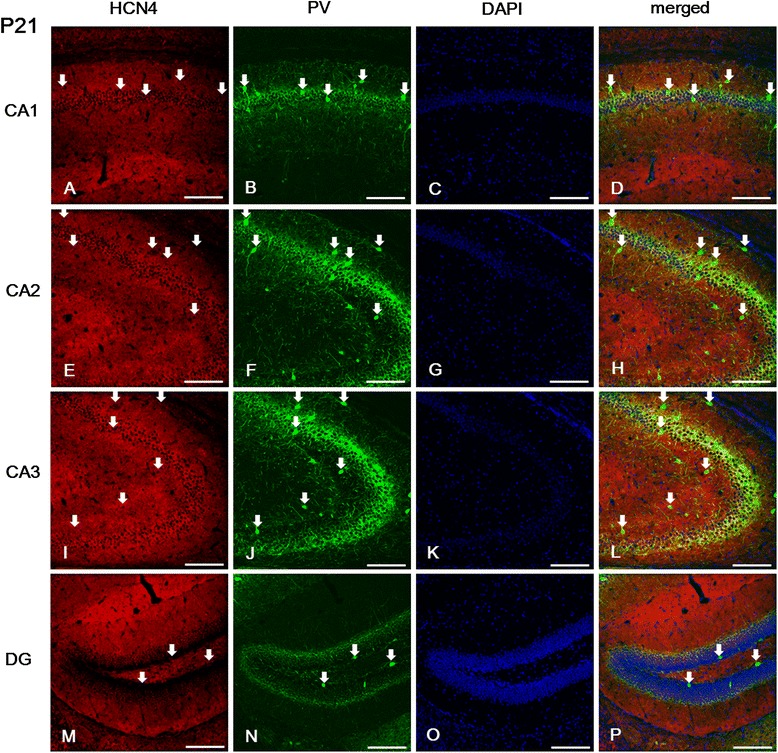
**Expression of HCN4 in PV-positive cells of the hippocampus at P21. (A-B, D-F, H-J, L-N, P)**: The images show HCN4 labeling in different fields of the hippocampus and the DG. **(A, E, I)**: Note that HCN4 expression was most prominent in the *sl-m* and *sp* of the CA1 **(A)** and CA2 **(E)**, and in the *sl* of the CA3 **(I)**; but it was absent from the *alveus*. In addition, most PV-immunopositive cells located in the *sp* of the CA1, the *so* and *sp* of the CA2, *sl*, *sp*, and *so* of the CA3, and the *hilus* of the DG also co-expressed HCN4. **A**-**B**, **D**-**F**, **H**-**J**, **L**-**N**, **P**: white arrows indicate PV-positive/HCN4-positive cells. **C**, **G**, **K**, **O**: DAPI staining. Scale bars = 20 μm.

The strongest labeling for HCN1 was observed in the SL-M and *alveus* of the CA (Figure 3A, E, I); its expression was gradually stronger in the SO, SP, and SR of the CA2 (Figure 3E) and CA3 (Figure 3I) compared to the CA1 (Figure 3A). The most prominent immunolabeling for HCN2 and HCN4 subunits was observed in the SL-M and SP of the CA; this labeling was particularly strong in the CA3 compared to the CA1 or CA2 (Figures 4A, E, I and 5A, E, I). Interestingly, HCN2 and HCN4 subunits were not expressed in the *alveus* (Figures 4A, E, I and 5A, E, I), which, on the other hand, showed the most intense expression for HCN1 (Figure 3A, 3I). Moreover, we observed a relatively intense labeling for HCN1 in the SR of the CA, particularly in the CA3 (Figure 3e and i), compared to the expression of HCN2 (Figure 4E, I) and HCN4 (Figure 5E, I). In addition, immunoreactivity for HCN4 (Figure 5I), but not for HCN1 (Figure 3I) or HCN2 (Figure 4I), was present in the SL of the CA3.

Next, we investigated the expression of each HCN subunit in interneurons, particularly in parvalbumin (PV)-positive GABAergic interneurons. In the CA area, we found the most intense labeling for PV in the CA3, and PV-immunopositive cells were mainly located in the SP of the CA (Figures 3, 4 and 5). A relatively moderate expression of PV was observed in the SO, and its expression was very weak in the SR, SL, and SL-M, compared to the SP (Figures 3, 4 and 5). Most PV-positive cells located in the SP and SO presented immunolabeling for HCN2 (Figures 4D, H, L) and HCN4 (Figure 5D, H, L) in the CA. However, PV-positive cells located in the SP of the CA1 did not show immunolabeling for HCN1 (Figure 3A, B, D), whereas those located in the SR or the SO were HCN1-positive (Figure 3A, B, D).

**Figure 6:**
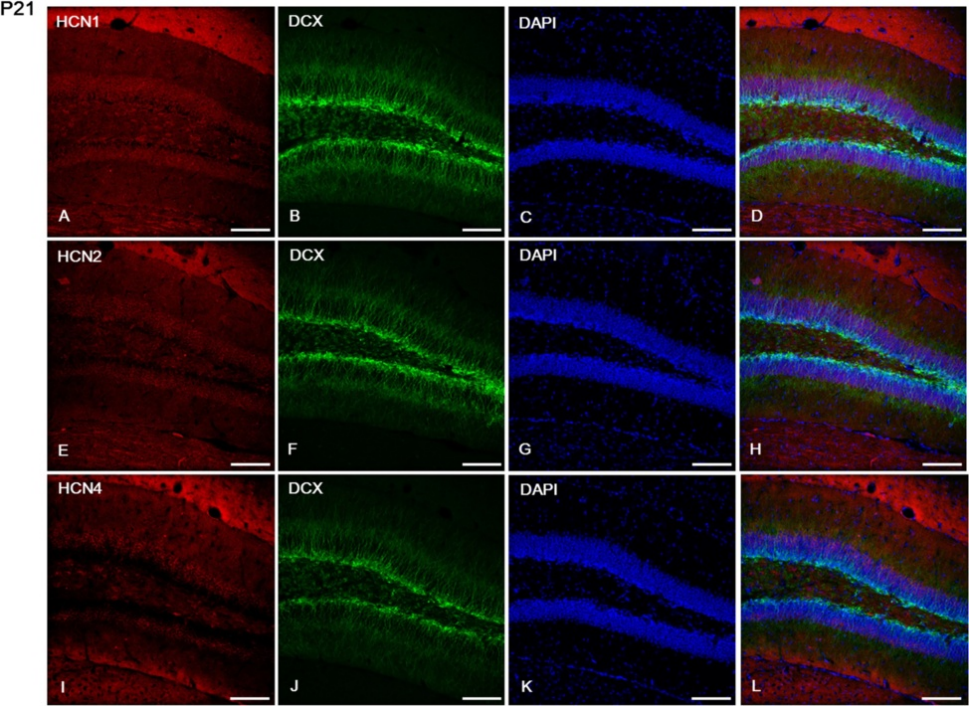
**Expression of HCN subunits in doublecortin (DCX)-positive cells of the dentate gyrus at P21. (A-L)**: These images show our results on double immunofluorescence for HCN subunits and DCX. In the DCX-immunopositive subgranular zone we did not find labeling for HCN1 **(A-D)**, HCN2 **(E-H)**, or HCN4 **(I-L)**. Scale bars = 20 μm.

In the *dentate gyrus*, immunolabeling for HCN1 was observed in the GCL and in a restricted population of cells of the *hilus* (Figure 3M-P). Immunoreactivity for HCN2 was strong throughout the *dentate gyrus*, but the most prominent expression was observed in the ML and *hilus* (Figure 4M-P). On the other hand, immunoreactivity for HCN4 was relatively low throughout the *dentate gyrus* (Figure 5M-P). HCN1- and HCN2-positive cells were located in the GCL (Figures 3M and 4M), but not in the subgranular zone (Figure 6A-H), which was labeled with doublecortin (DCX) as a marker of adult neurogenesis. HCN4-immunoreactive cells were generally located in the superficial part of the GCL, adjacent to the ML (Figure 5M), but not in the subgranular zone (Figure 6I-L), in a similar manner to HCN1 and HCN2 subunits. In addition, several PV-immunopositive interneurons showed HCN1 (Figure 3M-P), HCN2 (Figure 4M-P), and HCN4 (Figure 5M-P) expression in the GCL and *hilus*.

### Hippocampal expression of HCN channel subunits in the adult brain at P56

**Figure 7:**
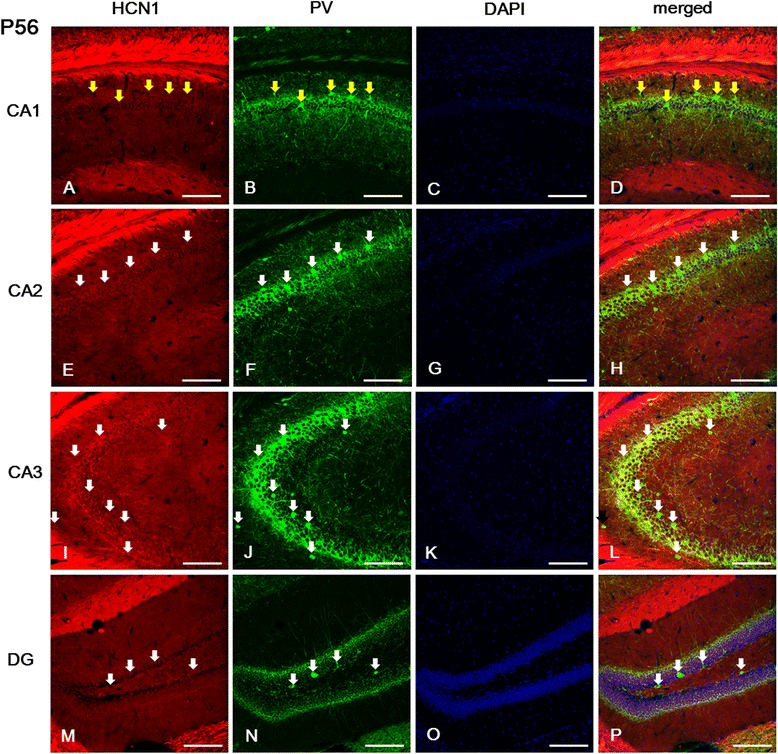
**Expression of HCN1 in PV-positive cells of the hippocampus at P56. (A, E, I)**: The strongest labeling for HCN1 was observed in the *sl-m* and *alveus* of the CA. Note also that its expression was gradually stronger in the *sp,* and *sr* of the CA2 **(E)** and CA3 **(I)** compared to the CA1 **(A)**. **(A, B, D)**: PV-positive cells located in the *sp* of the CA1 did not show HCN1 immunolabeling. On the other hand, in the CA2, CA3, and *hilus* of the DG most PV-immunopositive cells were labeled with HCN1. **A**-**B**, **D**: yellow arrows indicate PV-positive/HCN1-negative cells. **E**-**F**, **H**-**J**, **L**-**N**, **P**: white arrows indicate PV-positive/HCN1-positive cells. **C**, **G**, **K**, **O**: DAPI staining. Scale bars = 20 μm.

**Figure 8:**
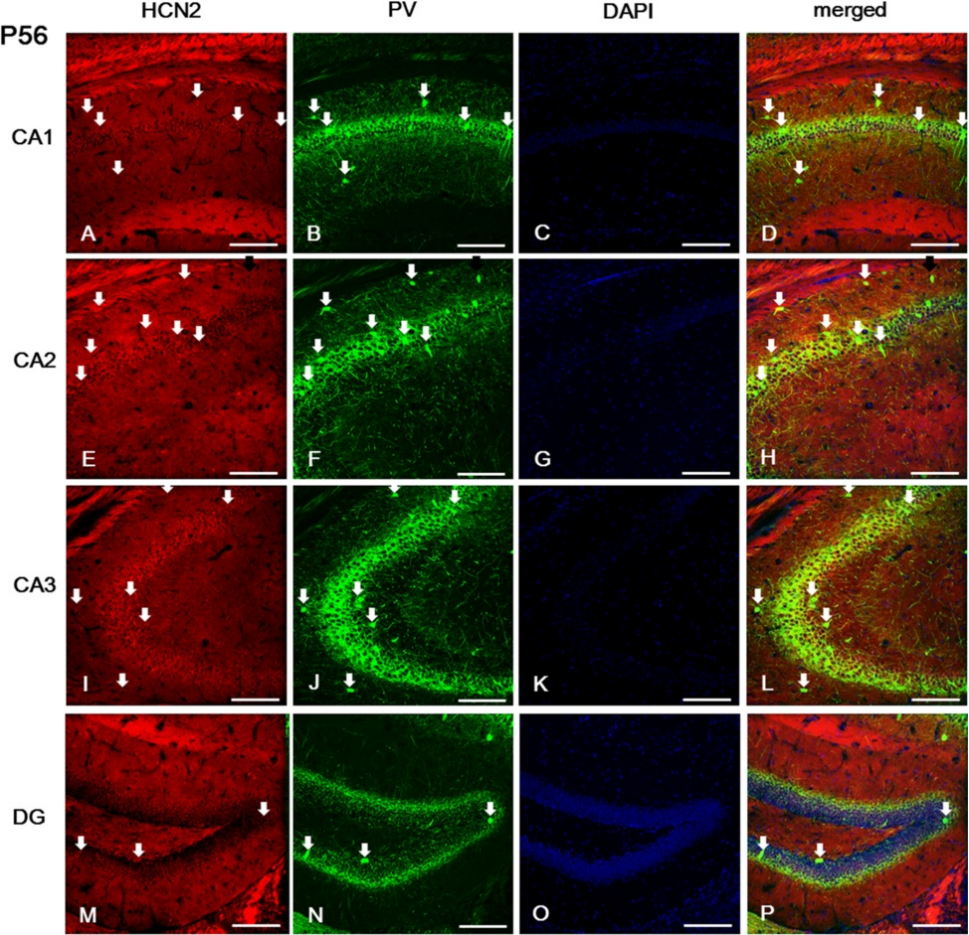
**Expression of HCN2 in PV-positive cells of the hippocampus at P56. (A-B, D, E-F, H-J, L-N, P)**: Most PV-immunopositive cells located in the CA1 **(A-B, D)**, CA2 **(E, F, H)**, CA3 **(I, J, L)**, and DG **(M, N, P)** were labeled with HCN2. **(A, E, I)**: HCN2 expression was most prominent in the *sl-m* of the CA1 **(A)**, as well as in the *sp* of the CA2 **(E)** and CA3 **(I)**. Interestingly, at this stage the *alveus* showed HCN2 labeling, contrasting with its expression pattern at P21. **A**-**B**, **D**, **E**-**F**, **H**-**J**, **L**-**N**, **P**: white arrows indicate PV-positive/HCN2-positive cells. **C**, **G**, **K**, **O**: DAPI staining. Scale bars = 20 μm.

**Figure 9:**
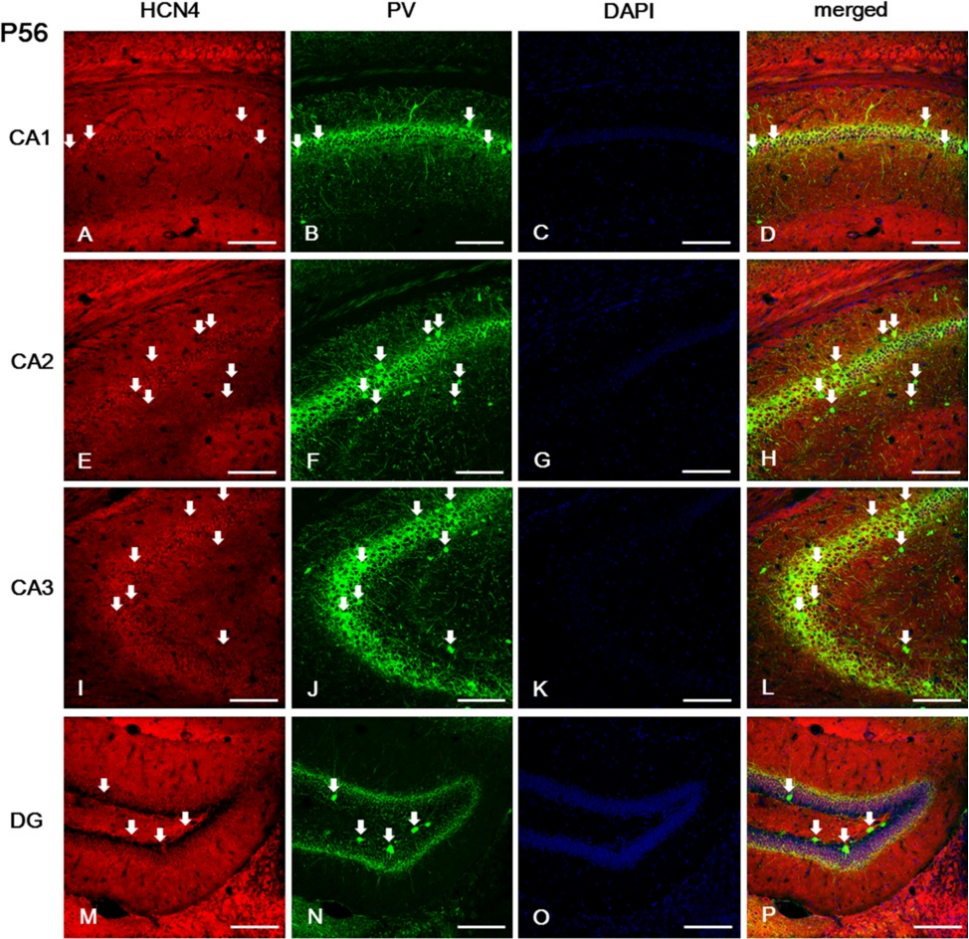
**Expression of HCN4 in PV-positive cells of the hippocampus at P56. (A-B, D-F, H-J, L-N, P)**: Most PV-immunopositive cells located in the *sp* of the CA1 **(A-B, D)**, the *so* and *sr* of the CA2 **(E, F, H)**, the *sl* and *sp* of the CA3 **(I, J, L)**, and the *hilus* of the DG **(M, N, P)** were also labeled for HCN4. **(A, E, I)**: Labeling for HCN4 was observed in the *sl-m* and *sp* of the CA1 **(A)**, CA2 **(E)**, and in the *sp* and *sl* of the CA3 **(I)**, as well as in the *alveus* of all CA fields. **A**-**B**, **D**, **E**-**F**, **H**-**J**, **L**-**N**, **P**: white arrows indicate PV-positive/HCN4-positive cells. **C**, **G**, **K**, **O**: DAPI staining. Scale bars = 20 μm.

In the CA field of the adult hippocampus, immunolabeled cells for HCN1, HCN2, and HCN4 subunits were found primarily in the SP, and in the SL-M layer (Figures 7, 8 and 9). Comparing the expression of these subunits, the immunolabeling for HCN4 was relatively lower than for HCN1 or HCN2. In the CA1 and CA2, immunoreactivity for HCN1 and HCN2 subunits was more prominent than for HCN4 in the SL-M; while in the SP the expression of HCN1 was relatively higher than that of HCN2 or HCN4 (Figures 7A, E, 8A, E and 9A, E). In the CA3, with the exception of the SL-M, the expression of all HCN subunits was more intense than in the CA1 or CA2 (Figures 7I-J, 8I and 9I). Interestingly, in the *alveus*, immunolabeling for HCN2 and HCN4 was relatively lower than for HCN1, whereas in earlier stages (i.e., P21) only HCN1 was observed in the *alveus* (Figures 7, 8, and 9).

We sought to examine next whether interneurons express any of the HCN subunits in adult hippocampus. Double immunofluorescence for HCN channel subunits and PV showed that HCN2 and HCN4 subunits were expressed in the PV-positive GABAergic interneurons located in the SO, SP, and SR layer of the CA (Figures 8A-L and 9A-L). However, HCN1 was not colocalized with PV immunolabeling in the SP layer of the CA1 (Figures 7A-D), but was colocalized with PV-positive cells in all subfields of the CA2 and CA3 (Figures 7E-L). In the *dentate gyrus*, PV-positive cells located in the GCL and *hilus* presented immunolabeling for all HCN subunits (Figures 7M-P, 8M-P and 9M-P). In addition, the immunolabeling for all of these subunits was primarily present in the GCL and *hilus*, in a similar manner to the pattern observed in P21 (Figures 7M, 8M and 9M). In the *dentate gyrus*, HCN1- and HCN2-positive cells were expressed throughout the GCL (Figures 7M-P and 8M-P), while HCN4-immunoreactive cells were more prominent in the superficial part of the GCL adjacent to the ML (Figure 9M-P). In a similar manner to the *dentate gyrus* at P21, none of the HCN subunits was expressed in the subgranular zone, suggesting that HCN channels are not involved in adult neurogenesis in the hippocampus. The intensity of immunostaining in the CA field and dentate gyrus for HCN1, HCN2, and HCN4 at P21 and P56 is summarized in Table 1.

**Table 1:** **Density of HCN isoform expression in the hippocampus at P21 and P56**

	**HCN1**		**HCN2**		**HCN4**	
	**3 weeks**	**8 weeks**	**3 weeks**	**8 weeks**	**3 weeks**	**8 weeks**
**CA1**						
SL-M	++	++++	++++	++++	++++	++
SR	+	-	-	+	-	+
SP	+	+	++	+	++	+
SO	-	-	-	+	-	+
Alveus	++++	++++	-	++	-	++
**CA2**						
SL-M	++	+++	++++	++++	++++	++
SR	+	-	-	+	-	+
SP	++	+	++	+	++	+
SO	+	-	+	+	+	+
Alveus	++++	++++	-	++	-	++
**CA3**						
SL-M	++	+++	++++	++	++++	++
SR	++	++	-	+	+	+
SL	-	-	-	-	++	-
SP	+++	+++	++++	+++	++++	++
SO	+	+	-	+	+	+
Alveus	++++	++++	-	+	+	+
**Dentate gyrus**						
ML	-	-	++	+	+	+
GCL	+	+	++	+	+	+
hilus	++	+	++	++	+	++

++++, very high expression; +++, high expression; ++, moderate expression; +, low expression; −, no detection.

## Discussion

The present study provides a map of the subunit- and cell-specific expression of HCN channels in the developing and adult hippocampus. We showed that immunolabeling for HCN1, HCN2, and HCN4 subunit was concentrated in distal dendrites of the pyramidal cells of the SL-M in the hippocampus, from P0 to adult. This suggests that HCN subunits may be assembled to form heteromeric channels to control dendritic functional integration in the hippocampus.

### Hippocampus and HCN channel

The hippocampus is known to play a critical role in learning and encoding memory such as temporal informative memory for spatial location [33]. In addition, many studies have demonstrated that different hippocampal subregions are involved in selectively distinct cognitive functions [34], suggesting that the hippocampus should not be considered as a single functional unit. Despite the remarkable influence of CA3 output pathways (through Schaffer collateral connections) on the CA1, which indicates that both regions are cooperating in certain cognitive functions [34], there are also important differences. For example, the CA1 is involved in processing of temporal object and spatial information, while the CA3 only mediates temporal memory for spatial information [33]. Moreover, CA3 function is associated with short-term memory, whereas the CA1 contributes to intermediate/long-term memory formation and consolidation [35,36]. In a previous study, Shin and Chetkovich [37] published that distal apical dendritic expression of HCN1 in the SL-M of the CA1 was initiated and controlled by direct inputs from the entorhinal cortex, but not the CA3, suggesting a possible dissociation between CA3 and CA1 in anatomical connections and function. Therefore, it will be necessary to conduct further developmental studies to elucidate the specific role and expression pattern of each HCN subunit in different hippocampal regions. Furthermore, it has been shown that postnatal adult neurogenesis primarily occurs in the dentate gyrus [38]. Our data showed that none of the HCN subunits were expressed in the subgranular zone of the dentate gyrus, which is the main location of postnatal neurogenesis. Thus, our data suggest that HCN subunits may not contribute to adult neurogenesis.

### Age-dependent and cell-specific expression of HCN channel in the hippocampus

HCN subunits were expressed in glutamatergic pyramidal neurons and in PV-immunopositive GABAergic interneurons in the hippocampus. Moreover, we found that during development of the hippocampus, the increase in expression of HCN1 was concomitant with a decrease in the expression of HCN2 and HCN4 subunits. These findings are consistent with the increased contribution of HCN1 to the Ih current in the adult hippocampus [3,39]. Therefore, the physiological changes in Ih currents that occur during hippocampal development may originate from the age-dependent expression of HCN subunits and their rearrangement during neurodevelopment, which may lead to differential neural and circuitry functions.

In addition, we found a HCN4-positive subpopulation of astrocytes in the SL-M, and in the border of the SL-M with the ML of the *dentate gyrus* at P7, but not after P21 (data not shown). This developmental stage-specific expression of HCN4 in astrocytes suggests an unexpected role for Ih currents in astrogliogenesis. Recently, Honsa et al. [40] reported that HCN4 subunits were not expressed in either the normal adult hippocampus or the ischemic injured-hippocampus, whereas HCN1 channels were found in many reactive astrocytes of hippocampus after ischemic injury [40]. Several lines of evidence have shown that the processes of astrogliogenesis and astrogliosis are controlled by similar mechanisms through the JAK-STAT signaling pathway, and differences in the mechanism controlling these two processes remain unknown [41-43]. However, taking into account previous reports and our data, we suggest that HCN4 channels may be involved primarily in early astrogliogenesis during development, while HCN1 channels may be implicated in adult astrogliosis related to injury. Overall, these data suggest that HCN channels may have roles in various types of neuronal/glial functions during development, and may be implicated in the modulation of brain cognitive functions, including learning and memory.

### Comparison of hippocampal HCN channel mRNA and protein distribution

In a previous study, Santoro and colleagues [17] reported differential expression of HCN1, HCN2, and HCN4 mRNA in the adult mouse hippocampus using in situ hybridization. Our data on HCN subunits protein immunolabeling was generally in agreement with this previous report on HCN mRNA expression. In fact, as in this previous report, we found expression of HCN1 and HCN2 in the SP of the CA and in the hilus of the dentate gyrus. Santoro et al. [17] also reported that HCN2 mRNA expression was relatively strong and HCN4 mRNA was not generally strong in the hippocampus compared to the expression of HCN1. Consistent with these data, we observed relatively stronger immunolabeling for HCN2 and weaker immunopositive signals for HCN4 in the CA and dentate gyrus of the adult mouse. However, there were some discrepancies between mRNA and protein expression of HCN1 in the SL-M; in fact, we found intense immunolabeling in the SL-M of the CA field, which contains dendritic processes, while mRNA expression was not strong in this area [17]. This suggests that HCN1 protein might be transported from neuronal somas to apical dendrites in the adult hippocampus.

### Clinical implications of HCN channel

Recently, several lines of evidence in animal and human studies have suggested that HCN channels may play a role in psychiatric conditions such as mood and anxiety disorders [18-20]. The loss of function by complete knockout of the HCN1 gene resulted in an abnormal behavioral phenotype that included the impairment of motor learning and memory in mice [44,45]. On the other hand, enhancement of the hippocampal activity by the deletion of the HCN1 gene induced anxiolytic and antidepressant behaviors via the activation of the BDNF-mTOR signaling pathway [46]. Moreover, a study in patients reported that a polymorphism in the HCN4 gene was implicated in mood-anxiety disorder phenotypes [47]. Finally, it has been shown that an imbalance between the expression of HCN1 and HCN2 subunits in the CA1 was involved in cognitive dysfunctions induced by drug addiction [48]. Although the mechanism by which the HCN channels have effects on cognitive function and mental disorders remains unclear, our findings showing age-dependent and cell type-specific expression of HCN channels may help to elucidate therapeutic approaches to modulate HCN channels.

## Methods

### Animals and tissue preparation

Adult C57BL/6 J mice were maintained in a 12-h light/dark cycle in a pathogen-free environment. The Institutional Animal Care and Use Committee at Kyungpook National University approved the research protocol. Females were mated with males in a timed schedule (from 8:00 p.m. to 08:00 a.m.). Females were checked for the presence of a vaginal plug at 08:00 a.m. the following morning. The day on which the vaginal plug was detected was defined as embryonic day 0.5 (E0.5), and the day of birth was considered as postnatal day 0 (P0). To harvest E14.5 embryonic brains, timed-pregnant dams were euthanized by cervical dislocation, the embryos were removed, and brains were dissected out and immersed overnight in 4% paraformaldehyde in phosphate-buffered saline (PBS). To harvest brains from early postnatal stages (i.e., P7), mouse pups were deeply anaesthetized by inhalation of isoflurane, and perfused transcardially with the same fixative. After cryoprotection, 12 μm-thick brain coronal sections were obtained on a cryomicrotome (Leica). For juvenile (P21) and adult (P56) mice, four male of each age were deeply anesthetized with an intraperitoneal injection of tribromoethanol (Avertin, 0.0125 mg/g of body weight). These animals were perfused through the aorta with PBS for 1 min, followed by an ice-cold fixative containing 4% paraformaldehyde in PBS (pH 7.4), for 15 min. Brains were immediately removed after fixation, and coronal 12-μm-thick sections were obtained on a cryomicrotome.

### Antibody selection and specificity

Rabbit polyclonal antibodies against HCN1 (APC-056), HCN2 (APC-030), and HCN4 (APC-052) were obtained from Alomone Labs (Jerusalem, Israel). The manufacturer confirmed the specificity of each antibody using Western blot. Each antibody yielded a single specific band of the expected molecular weight using extracts of rodent brain membrane proteins. We also performed western blot analysis of the mouse brain proteins to confirm the specificity of antibodies and we detected a specific band at the expected molecular weight. In addition, we conducted pre-absorption test to assess the specificity of the antibodies in immunofluorescence of mouse brain tissue. Specific peptides for HCN1 or HCN2 were added to each primary antibody working solution using a 1:1 (antibody:peptide) ratio, while purified HCN4 protein was added on a 1:3 (antibody:protein) ratio to the HCN4 antibody solution. Our pre-absorption tests confirmed the specificity of the labeling observed in our immunofluorescence experiments since no labeling for HCN1, HCN2, or HCN4 was observed in the pre-absorbed brain sections (data not shown).

### Immunofluorescence and image analysis

Brain tissue sections were rinsed in PBS, and permeabilized for 1 h at room temperature with 0.1% Triton X-100 in PBS containing 5% normal goat serum. Sections were then incubated for 48 h at 4 °C in the primary antibody (1:100 dilution for each HCN subunit) in PBS containing 5% normal goat serum. After rinsing in PBS (three times, 10 min each), the sections were incubated with an Alexa Fluor 594-conjugated goat anti-rabbit IgG (Invitrogen) diluted 1:250 in PBS containing 5% normal goat serum, for 2 h at room temperature. Finally, the sections were rinsed in PBS (three times, 10 min each), and coverslipped with Vectashield (Vector Laboratories). For double immunofluorescence experiments, each of the HCN subunit antibodies was combined with an anti-mouse doublecortin (1:1000, DCX; Santa Cruz, SC-8066), or an anti-mouse parvalbumin (1:1000, PV; Millipore, MAB1572) antibody. After incubation with the primary antibody overnight, the sections were incubated with an Alexa Fluor 488-conjugated goat anti-mouse IgG (1:200) in PBS containing 5% normal goat serum, for 2 h at room temperature.

Images were obtained on a Zeiss (Thornwood, NY) confocal microscope using x5 and x20 objectives lenses. For each antigen, all brain sections were processed identically, and images were obtained using the same microscope parameters. The relative intensity of immunopositive signals for HCN1, HCN2, and HCN4 subunits in the hippocampus was measured using ImageJ software [49]. HCN immunolabeling of GFAP-positive or PV-positive cells was assessed during confocal microscope imaging.

## Abbreviations

CA: Cornu Ammonis
DCX: Doublecortin
E: Embryonic day
GCL: Granule cell layer
GFAP: Glial fibrillary acidic protein
HCN: Hyperpolarization-activated cyclic nucleotide-gated
IZ: Intermediate zone
ML: Molecular layer
P: Postnatal day
PV: Parvalbumin
SL: Stratum lucidum
SL-M: Stratum lacunosum-moleculare
SO: Stratum oriens
SP: Stratum pyramidale
VZ: Ventricular zone
